# The Role of C-Reactive Protein in the Early Prediction of Serious Pancreatic Fistula Development after Pancreaticoduodenectomy

**DOI:** 10.1155/2018/9157806

**Published:** 2018-01-28

**Authors:** Fatma Umit Malya, Mustafa Hasbahceci, Yunus Tasci, Huseyin Kadioglu, Mehmet Guzel, Oguzhan Karatepe, Kemal Dolay

**Affiliations:** ^1^Bezmialem Vakif University, Istanbul, Turkey; ^2^Medical Park Fatih Hospital, General Surgery Clinic, Istanbul, Turkey; ^3^Medicana Hospital, Istanbul, Turkey; ^4^Memorial Hospital, Istanbul, Turkey

## Abstract

**Introduction:**

Despite recent advances in surgical techniques, pancreatic fistulas are common. We aimed to determine the role of C-reactive protein in the prediction of clinically relevant fistula development.

**Materials and Methods:**

Data from patients who underwent pancreaticoduodenectomy between 2012 and 2015 is collected. Postoperative 1st, 3rd, and 5th day (POD1, POD3, and POD5) C-reactive protein (CRP) levels, postoperative pancreatic fistula (POPF) development, other complications, length of hospital stay, and mortality were recorded.

**Results:**

Of 117 patients, 43 patients (36.8%) developed complications (including fistulas). Of the patients developing fistulas, 21 (17.9%) had POPF A, 2 (1.7%) had POPF B, and 7 (6.0%) had POPF C. POD5 CRP and POD3 CRP were shown to be significantly correlated with mortality and development of clinically relevant POPF (*p* = 0.001 and *p* = 0.0001, resp.) and with mortality (*p* = 0.017), respectively. The development of clinically relevant POPFs (B and C) could be predicted with 90% sensitivity and 82.2% specificity by POD5 CRP cut-off level of 19 mg/dL and with 100% sensitivity and 63.6% specificity by the difference between POD5 and POD1 CRP cut-off level of >2.5 mg/dL.

**Conclusion:**

CRP levels can effectively predict the development of clinically relevant pancreatic fistulas.

## 1. Introduction

Pancreaticoduodenectomy (PD) is a procedure that is used to treat diseases of the periampullary area. It is a complex and invasive method that requires a high degree of technical skill. Due to the anatomical complexity of the area, the fragility of the soft pancreatic tissue, and the risk of pancreatic fluid leakage, this procedure is predominantly performed in high-volume centers. Despite recent important advances in surgical techniques, technology, and perioperative care, mortality and morbidity rates remain high (5% and 35–60%, resp.), even in experienced centers [1]. Pancreas-specific complications, particularly pancreatic fistula, bleeding, and intraabdominal collection, are the leading causes of mortality and morbidity following pancreaticoduodenectomy. As a result of these complications, intraabdominal infection and bleeding can occur, causing an increased risk of mortality and prolonged length of hospital stay and, thereby, increased costs. Among these complications, postoperative pancreatic fistula (POPF) is the most serious [2–6]. Many studies have investigated the causes of the development of POPF, including the influence of intraoperative findings, particularly amount of bleeding, properties of the pancreas, and diameter of the pancreatic duct. Additionally, many studies have examined the effects of various factors, including patient age, body mass index, and indications for the surgical treatment including malignant and benign etiologies. It has been found that poor pancreatic quality, small pancreatic duct diameter, and obesity are associated with high rates of the development of POPF. There have been efforts to predict the development of serious POPF by using clinical scoring systems in the postoperative period. It has been thought that such approaches may help physicians to minimize additional morbidity and mortality [7].

C-reactive protein (CRP) is an acute phase reactant with a half-life as short as 19 hours. Therefore, it is conveniently used for the assessment of disease status and inflammatory response during the postoperative healing process [8]. In 1976, Fischer et al. [9] were the first to show the role of CRP in the prediction of postoperative inflammatory complications. Consequently, several studies have examined the role of CRP in the prediction of postoperative leakage and inflammatory complications after colorectal and gastroesophageal surgery [10–12]. However, there is controversy regarding the role of CRP in predicting the development of POPF and its cut-off value, because it is clear that surgical trauma and several inflammatory complications can alter its level [13, 14]. The prediction of serious pancreatic fistula development and other inflammatory complications following pancreatectomy is one of the important aims of the postoperative care. Therefore, the present study aimed to determine the significance of CRP levels for predicting clinically relevant fistula development after PD performed for periampullary tumors.

## 2. Materials and Methods

### 2.1. Ethics

This study conforms to the ethical guidelines of the World Medical Association Declaration of Helsinki with regard to ethical principles for medical research involving human subjects. The study was approved by the local ethical committee (12072017-12387). The study was registered with http://www.researchregistry.com with an ID number of 2604. All included patients provided written informed consent for the surgery and the usage of clinical data.

### 2.2. Patients

We retrospectively reviewed the data of all patients who underwent PD between 2012 and 2015. The data reviewed are as follows: demographic data with regard to age, gender, body mass index calculated as weight in kilograms divided by the square of height in meters (kg/m^2^), and previous history of acute pancreatitis and diabetes mellitus. Diabetes mellitus was considered in the presence of at least one diagnostic criteria: (1) hemoglobin A1c (A1c) ≥ 6.5% or (2) fasting plasma glucose ≥ 126 mg/dL (fasting is defined as no caloric intake for at least 8 hours) or (3) 2-hour plasma glucose ≥ 200 mg/dL during an oral glucose tolerance test or (4) random plasma glucose ≥ 200 mg/dL in a patient with classic symptoms of hyperglycemia.

The following data were recorded from each patient: postoperative 1st, 3rd, and 5th day (POD1, POD3, and POD5) CRP levels (mg/L), the development of POPF and other complications, length of hospital stay, and mortality. In addition, preoperative and operative findings, as well as postoperative follow-up findings, were recorded. During the operation, subjective evaluation of the pancreas quality by the attending surgeon as hard, moderate, or soft; the pancreatic duct diameter as ≤3 mm and >3 mm; the amount of bleeding as mL; and operation time (minutes) were noted. In the postoperative period, complications, time of drain removal, length of hospital stay, and requirement for additional interventions or surgery were noted.

### 2.3. Surgical Technique

All patients who underwent the same operative technique are briefly described herein. The organs that were excised during PD included the gallbladder, the common hepatic duct, the head of the pancreas, the duodenum (except for a 3-4 cm portion near the bulb), and approximately 10 cm of the proximal jejunum. The pancreas was divided with a scalpel. The duodenum was transected approximately 3-4 cm distal to the pyloric ring for pylorus-preserving pancreatectomy, and the stomach was transected approximately 2-3 cm proximal to the pyloric ring at the antrum in classical pancreatectomy. We performed pancreaticojejunostomy via duct-to-mucosa method with interrupted sutures of 4-0 PDS-II (polydioxanone; Johnson & Johnson Co., United States). A stent tube was inserted into the pancreatic duct, except for those patients with a remarkably dilated main pancreatic duct. The jejunal wall and the pancreatic stump were adhered together with one-layer sutures. An end-to-side hepaticojejunostomy was then performed 5–10 cm distal from the pancreaticojejunostomy.

Finally, end-to-side duodeno- or gastrojejunostomy was performed to place the stomach and the duodenum into a straight vertical line for an antecolic route. Closed drains were inserted behind the hepaticojejunostomy and at the upper side of the pancreaticojejunostomy (Blake® drain, Ethicon, Somerville, NJ, United States).

### 2.4. Definition of Pancreatic Fistula

The diagnosis of POPF was determined according to the definitions and classifications stated in the International Study Group for Pancreatic Fistula (ISGPF) guidelines [5]. Namely, any measurable volume of the drain fluid on or after POD3 with an amylase content more than 3 times of the upper normal serum value (>127 IU/ml) was defined as POPF. POPFs were then classified as follows: grade A transient fistula or biochemical leakage—the presence of a biochemical leakage is defined as the pancreatic amylase output higher than three times of the upper normal serum level, from the drain placed during surgery. The patient is fed orally and remains clinically well.

A clinically significant grade B pancreatic fistula is present in the presence of increased amylase activity in the fluid from any drain in association with a clinically relevant condition necessitating any change in the management of the expected postoperative pathway [5]. In the presence of grade B POPF, whenever organ failure or clinical instability develops, this situation is named as grade C POPF usually necessitating reoperation and prolonged hospitalization and stay in intensive care units [5].

In the present study, grades B and C were defined as the clinically relevant POPF. Patients with the clinically relevant fistula were grouped as group 1 and the other patients were grouped as group 2.

### 2.5. Perioperative Care

Ceftriaxone (Forcef, 1 g, IV, Bilim, Istanbul, Turkey) and metronidazole (Flagyl, 0.5%, 100 mL, IV infusion, Eczacibaşi, Istanbul, Turkey) were administered immediately before the surgery and every 3 h during the surgery according to the university infection committee recommendations. All patients were administered with antibiotics and proton pump blockers routinely until POD3. Oral intake was started routinely at the postoperative fifth day, unless there were postoperative complications preventing oral intake. Blood sugar levels were routinely monitored and maintained between the blood sugar levels of 70 mg/dL and 200 mg/dL. The amylase content in the drain fluids (IU/mL) was measured on PODs 3 and 5.

Continuous low-pressure suction was applied to the abdominal drains to minimize the amount of fluid in the postoperative surgical area. Abdominal drains were removed in patients without POPFs on POD 6, or they were replaced as needed until the POPFs were resolved. None of the patients routinely received octreotide.

### 2.6. Statistical Analysis

The development of clinically relevant POPF was identified as the main variable. Mortality and the length of hospital stay were other secondary outcomes. Statistical analysis was performed using the Statistical Package for Social Sciences (SPSS) version 22.0 (IBM SPSS, New York, USA). Normally distributed continuous variables were expressed as mean ± standard deviation, and median was used for nonnormally distributed continuous variables. Categorical variables were expressed as frequencies and percentages. Characteristics of the groups were compared using the *t*-test for normally distributed continuous variables and Mann–Whitney *U* test for continuous variables without normal distribution. Pearson's chi-square tests were used to compare categorical variables. Spearman's correlation analysis was performed between CRP values and their changes with the development of transient and clinically relevant POPF, mortality, and the length of hospital stay. Mann–Whitney test was also used to analyze the association between CRP values and their changes with mortality.

Receiver-operating characteristic (ROC) curves with the area under the curve (AUC) values were used to evaluate cut-off values and to determine changes over time. These ROC curves were used to calculate CRP cut-off values for predicting clinically relevant pancreatic fistula development. The effect of these values on the length of hospital stay was analyzed. The statistical results were presented at a 95% confidence interval. The differences were considered statistically significant if the *p* value was less than 0.05.

## 3. Results

A total of 117 patients who underwent pancreatectomy were included in this study. Of these patients, 71 (60.6%) were male and 46 (39.3%) were female. The mean age of the patients was 60.7 ± 13.3 years.  Table 1 shows the demographic and clinicopathological features of the patients. Amylase levels in drain fluid and serum of the patients were significantly higher in group 1. Regarding their postoperative pathological diagnoses, adenocarcinoma of the head of the pancreas (51.2%) and intrapapillary mucinous neoplasm of the pancreas (25.6%) were the most common pathologies (Table 1). The mean length of hospital stay was 11.3 ± 5.9 days. Nine of the patients died during the postoperative one month with a total mortality rate of 7.7%. Overall complications and the complications leading to mortality are shown in Table 2.

Grade A, B, and C fistulas were observed in 21 (17.9%), two (1.7%), and seven (6.0%) patients, respectively. Therefore, clinically relevant fistulas (grades B or C) were observed in nine patients (group 1) (7.7%). Groups 1 and 2, the patients with and without clinically relevant POPF, respectively, were similar with regard to demographic and clinicopathological features (*p* > 0.05 for all). Grouping based on the diameter of the pancreatic duct as ≤3 mm and <3 mm revealed no significance (*p* = 1.000).

**Figure 1:**
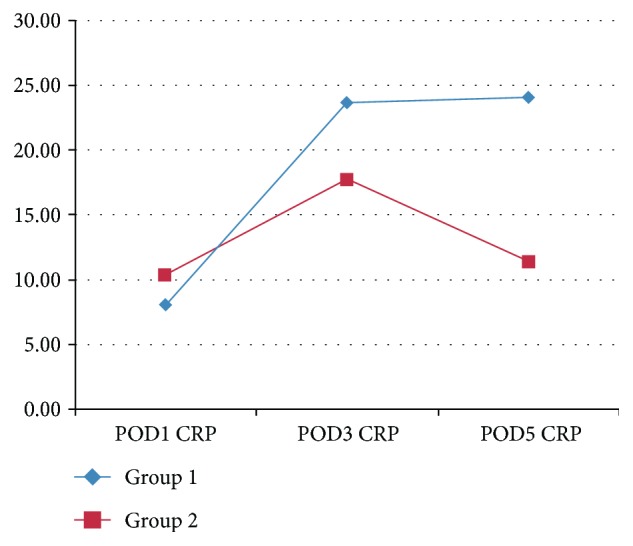
Changing pattern of CRP levels in patients with (group 1) and without POPF (group 2) from POD1 to POD5.

CRP values and the changes in CRP values with regard to the postoperative days were given in Table 3. CRP values at the POD3 were the highest compared with other days. There was a significant difference in POD5 CRP level between the groups (*p* = 0.0001). Additionally, the lack of the decrease from POD3 to POD5 in CRP levels as observed in patients without POPF (group 2) was not seen in patients with POPF (group 1) (Figure 1).

Spearman's correlation analysis revealed that POD3 and POD5 CRP were correlated with mortality and POD5 CRP was correlated with the development of clinically relevant POPF (Table 4).

In addition, there were significant correlations between POD5 CRP, Δ (POD5-POD1) CRP, and Δ (POD5-POD3) CRP and clinically relevant POPF compared with transient fistula (Table 5).

Among the CRP levels on the postoperative 1st, 3rd, and 5th days (POD1, 3, and 5), only POD3 and POD5 CRP levels were also shown to be significantly associated with mortality (Table 6).

The analysis of the changing pattern in CRP levels between the postoperative days revealed that the difference between POD1 and POD3 CRP levels (Δ POD3-POD1) and POD1 and POD5 CRP levels (Δ POD5-POD1) were also significantly associated with the development of mortality (Table 6).

To determine cutoff values of CRP values, ROC analysis using the sensitivities and specificities based on the diagnosis of clinically relevant fistula revealed that CRP level of >19 mg/dL at POD5 and CRP changes of >2.5 mg/dL between POD5 and POD1 (Δ POD5-POD1) were significantly associated with the prediction of clinically relevant POPF (Table 7).

## 4. Discussion

The development of clinically relevant pancreatic fistula after PD can cause postoperative sepsis and bleeding complications, leading to serious morbidity and mortality [4, 15].

Moreover, pancreatic fistula development can also influence cancer recurrence [16]. It is important to restrict the growth of pancreatic fistulas, and therefore, it is important to detect pancreatic fistula development in the early stages and to predict patients who are likely to develop serious pancreatic fistula. This will allow for the proper monitoring of high-risk patients, insertion of proper drainage, aggressive treatment of infective complications, proper manipulation of complications with sufficient nutritional support, prevention of clinical deterioration, and, most importantly, prevention of mortality.

Patients with clinically relevant postoperative pancreatic fistula (POPF) have a high risk of mortality due to sepsis and vascular erosions. Mortality is also high in cases who undergo reoperation and in cases who undergo complementary pancreatic resection [17, 18]. The International Study Group for Pancreatic Fistula (ISGPF) definition of pancreatic fistula is widely accepted. However, this definition is rather retrospective and, therefore, does not allow for clinical prediction. Because of this limitation, ongoing studies aim to identify parameters that can be used to predict clinical course in these patients. Further, as grade B and C fistulas are defined as those that have clinically serious consequences, they are generally considered as clinically relevant fistulas [19]. In the present study, we also accepted grade B and C fistulas as clinically relevant, and our analysis for prediction of fistula development included these groups. In our study, 17.9% of fistulas were grade A, 1.7% was grade B, and 5.98% were grade C. Therefore, the rate of clinically relevant fistula in our current study was 7.68%, which is consistent with the literature.

There are many studies related to the prediction of clinically relevant pancreatic fistula development. Kawai et al. [20] reported that fistula development could be predicted by male sex, intraoperative bleeding greater than 1000 cc, soft pancreas consistency, and postoperative 1st day drain fluid amylase level above 4000 IU/L. In another study, the same researchers stated that other independent parameters for predicting fistula development included serum albumin level below 3.0 g/dL and postoperative 4th day leukocyte count above 9800 mm^3^ [21]. Yamamoto et al. [22] proposed a scoring system that included pancreatic duct diameter, relationship with the portal vein, sex, intra-abdominal fat thickness, and some diagnostic parameters. In addition to preoperative and intraoperative parameters, various postoperative parameters have also been studied with the aim of identifying patients who are likely to develop serious fistulas and who require close monitoring.

Studies have shown that CRP levels can predict inflammatory complications and anastomosis leakage after colorectal surgery [23, 24]. Murakami et al. found that patients with soft pancreas consistency had elevated CRP levels, and they hypothesized that this property could be related with fistula development [25]. Several studies have examined the release profile of CRP in the postoperative period [26] and have found that CRP reaches peak levels at the 48th hour after surgery, after which it tends to decline, dropping to half of its peak level on the 5th postoperative day. Persistently elevated levels or increases after the 48th postoperative hour may indicate an inflammatory pathology.

Following pancreatectomy, elevation of CRP levels in the early period and persistently elevated levels may occur due to pancreatitis, tissue ischemia, tissue necrosis due to anastomotic leakage, wound site infection (developing due to ischemia at the wound site), and bacterial infection. Ischemia or intravascular lipopolysaccharide release can trigger the release of interleukin 6, which is the most important stimulant of CRP release. Once released, CRP binds to phosphocholine and recognizes foreign pathogens. CRP also activates the complement system and phagocytic cells. Through this pathway, the primary immune response is activated before the systemic inflammatory response is initiated [27]. Welsch et al. [13] reported that the persistence of elevated CRP levels on the 4th postoperative day could play an important role in predicting development of inflammatory complications after pancreas surgery. Hiyoshi et al. [28] stated that fistula development could be predicted by postoperative drain fluid amylase levels, CRP values, and body temperature; they also emphasized the importance of monitoring for bleeding complications [29]. In our current study, we found that POD5 and POD3 CRP levels could predict serious fistula development and/or mortality. POD5 CRP level and the changes between POD3 and POD5 CRP levels [Δ (POD5-POD1) CRP and Δ (POD5-POD3) CRP] may help physicians to differentiate clinically relevant POPF from transient pancreatic fistula. Cut-off values of 19 mg/dL for POD5 CRP level had high sensitivity and specificity for predicting clinically relevant fistula. Similarly, a difference of greater than 2.5 mg/dL between POD1 and POD5 CRP levels was also effective in predicting serious fistula. Therefore, interval measurement of postoperative CRP levels may help physicians to predict the development of POPF after PD. Additionally, some preventive measures including early imaging techniques, that is, computed tomography, evaluation of antibiotic treatment, use of somatostatin analogues, total parenteral or enteral nutrition or blood products, and percutaneous drainage in the presence of intra-abdominal collections may be considered in these patients.

The major limitations of our study include the relatively small number of patients and the evaluation of CRP as the only inflammatory parameter. Nonetheless, we obtained significant results. Further interpretations may be reached with larger-scale studies with prospective designs that can evaluate the present data for validity and reliability.

In conclusion, our results suggest that CRP plays an important role in predicting clinically relevant fistula development. Our calculated cut-off values may provide guidance in detecting patients who are likely to develop serious fistula. Further, it may be possible to detect serious complications and intervene in the early stages with close and aggressive monitoring of these patients. This will also help to reduce mortality, which is the most important goal.

## Figures and Tables

**Table 1:** Demographic and clinicopathological features of the patients.

Features	Overall (*n* (%))	Group1 (*n* (%))	Group 2 (*n* (%))	*p*
*N* (%)	117	9 (7.7)	108 (92.3)	
Age (mean ± SD) (year)	60.7 ± 13.3	61 ± 12	60.6 ± 13	0.586
Male/female	71/46	7/2	64/44	0.184
BMI (kg/m^2^)	23 ± 3.7	22 ± 3.7	23 ± 3.7	0.186
Preoperative diabetes	29 (24.7)	2 (22.2)	27 (25)	0.286
Pancreatitis history	15 (12.8)	1 (11.1)	14 (12.9)	0.483
Amylase in drain fluid (POD3) (U/mL)^μ^	1281.1 ± 5242.4 (41)	946.7 ± 1638.4 (150)	1312.3 ± 5462.2 (34)	0.007
Amylase in drain fluid (POD5) (U/mL)^μ^	1395.0 ± 5539.2 (22)	5551.2 ± 120 26.1 (330)	1006.6 ± 4417 (20)	0.0001
Serum amylase (POD3) (U/mL)^μ^	37.7 ± 42.6 (22)	60.6 ± 76.4 (30)	35.6 ± 37.9 (22)	0.234
Serum amylase (POD5) (U/mL)^μ^	40.6 ± 124.1 (23)	165.9 ± 414.3 (25)	28.8 ± 25.4 (22)	0.145
Quality of pancreas				
Soft	34 (29.1)	2	31	0.993
Moderate	46 (39.3)	4	42	
Hard	37 (31.6)	3	34	
Diameter of pancreatic duct (mm)	4.66 ± 2.15	4.70 ± 2.16	4.65 ± 2.15	0.949
Pathological diagnoses				
Adenocarcinoma of the head of the pancreas	60 (51.2)	3 (33.3)	57 (52.7)	0.08
IPMN	30 (25.6)	1 (11.1)	29 (26.8)	0.152
Ampullary adenocarcinoma	10 (8.5)	3 (33.3)	7 (6.48)	0.02
Cholangiocarcinoma	8 (6.8)	1 (11.1)	7 (6.48)	0.482
Malignant neuroendocrine tumor	3 (2.5)	1 (11.1)	2 (1.85)	0.563
Others	6 (5.1)	—	6 (5.55)	0.456
Blood loss (ml)	300 ± 50	350 ± 50	300 ± 50	0.286
Operation time (min)	345.9 ± 70	357.7 ± 73	344.8 ± 69	0.573
Length of hospital stay (day)	11 ± 5	11 ± 5	10 ± 4	0.053
Morbidity	43 (36.7)	9	34	0.0001
Mortality	9 (7.7)	5	4	0.0001

^μ^Mean ± standard deviation (median).

**Table 2:** Complications. The number of complications leading to mortality is given in parentheses.

Complications	Overall (*n*)	Group 1 (*n*)	Group 2 (*n*)
Surgical side infections	28	4	24
Pneumonia	1 (1)	—	1 (1)
Cardiac complications	1 (1)	—	1 (1)
Bleeding	5 (3)	3 (3)	2
Intra-abdominal abscess	2 (2)	2 (2)	
Hepaticojejunostomy leakage	2 (2)		2 (2)
Delayed gastric emptying	1	—	1
Chylous fistula	2	—	2
Gastroenteric anastomosis leakage	1	—	1
Total	43	9	34

**Table 3:** CRP values and the changes according to the postoperative days.

Parameter	Overall	Group 1	Group 2	*p*
*N* (%)	117	9 (7.7)	108 (92.3)	
POD1CRP	10.3 ± 7.2	8.10 ± 5.3	10.4 ± 7.3	0.331
POD3 CRP	18.3 ± 10.0	23.7 ± 9.9	17.8 ± 9.9	0.080
POD5 CRP	12.5 ± 9.6	24.1 ± 8.6	11.4 ± 9.0	0.0001
Δ POD3-POD1	8.1 ± 11.4	15.6 ± 11.0	7.4 ± 11.3	0.041
Δ POD5-POD1	2.2 ± 11.9	16.0 ± 9.6	1.0 ± 11.3	0.0001
Δ POD5-POD3	−5.9 ± 8.2	0.4 ± 10.8	−6.5 ± 7.7	0.045

**Table 4:** Correlation analysis of CRP values with clinically relevant POPF and mortality.

Variable	Correlation coefficient rho (*ρ*)/*p*	POPF	Mortality
POD1 CRP	Rho (*ρ*)	−0.090	0.054
	*p*	0.333	0.561
POD3 CRP	Rho (*ρ*)	0.163^∗^	0.220^∗∗^
	*p*	0.080	0.017
POD5 CRP	Rho (*ρ*)	0.340	0.293
	*p*	0.0001	0.001
Δ (POD3-POD1) CRP	Rho (*ρ*)	0.189^∗∗^	0.170^∗∗^
	*p*	0.041	0.067
Δ (POD5-POD1) CRP	Rho (*ρ*)	0.342^∗∗^	0.217^∗∗^
	*p*	0.0001	0.019
Δ (POD5-POD3) CRP	Rho (*ρ*)	0.186^∗^	0.115
	*p*	0.044	0.217

^∗^Correlation is significant at the 0.05 level (2-tailed). ^∗∗^Correlation is significant at the 0.01 level (2-tailed).

**Table 5:** Correlation analysis of CRP values with transient fistula (*n* = 21) and clinically relevant POPF (*n* = 9).

Variable	Correlation coefficient rho (*ρ*)/*p*	Transient versus clinically relevant POPF
POD1 CRP	Rho (*ρ*)	−0.313
	*p*	0.086
POD3 CRP	Rho (*ρ*)	−0.050
	*p*	0.788
POD5 CRP	Rho (*ρ*)	0.409^∗∗^
	*p*	0.022
Δ (POD3-POD1) CRP	Rho (*ρ*)	0.116^∗∗^
	*p*	0.535
Δ (POD5-POD1) CRP	Rho (*ρ*)	0.444^∗∗^
	*p*	0.012
Δ (POD5-POD3) CRP	Rho (*ρ*)	0.471^∗∗^
	*p*	0.007

^∗∗^Correlation is significant at the 0.01 level (2-tailed).

**Table 6:** Association of CRP values and their changes according to the postoperative days with mortality.

Parameter	Overall	Patients without mortality	Patients with mortality	*p*
*N* (%)	117	108 (92.3)	9 (7.7)	
CRP POD1	10.3 ± 7.2	10.2 ± 7.3	11.0 ± 6.3	0.559
CRP POD3	18.3 ± 10.0	17.6 ± 9.7	26.9 ± 10.8	0.018
CRP POD5	12.5 ± 9.6	11.5 ± 8.8	24.6 ± 11.2	0.002
Δ POD3-POD1	8.1 ± 11.4	7.5 ± 11	15.9 ± 14.3	0.067
Δ POD5-POD1	2.2 ± 11.9	1.3 ± 11.1	13.6 ± 15.6	0.020
Δ POD5-POD3	−5.9 ± 8.2	−6.2 ± 7.2	−2.3 ± 16.1	0.215

**Table 7:** ROC analysis with maximum combination of sensitivity and specificity of CRP values and their changes to predict clinically relevant POPF.

Parameter	Value	*p*	AUC	Sensitivity	Specificity	95% CI	
						Lower bound	Upper bound
POD1 CRP	>5.5	0.335	0.407	70	27.1	0.226	0.588
POD3 CRP	>22.5	0.080	0.668	70	71	0.508	0.828
POD5 CRP	>19	0.0001	0.851	90	82.2	0.742	0.959
Δ POD3-POD1	>7.5	0.042	0.695	80	58.9	0.546	0.844
Δ POD5-POD1	>2.5	0.0001	0.853	100	63.6	0.770	0.937
Δ POD5-POD3	> −7.5	0.045	0.692	90	42.1	0.527	0.857
